# COVID-19 in adults with dementia: clinical features and risk factors of mortality—a clinical cohort study on 125 patients

**DOI:** 10.1186/s13195-021-00820-9

**Published:** 2021-04-10

**Authors:** Agathe Vrillon, Elsa Mhanna, Clément Aveneau, Manon Lebozec, Lina Grosset, Diane Nankam, Fernanda Albuquerque, Raphaelle Razou Feroldi, Barbara Maakaroun, Iana Pissareva, Dalenda Cherni Gherissi, Julien Azuar, Véronique François, Claire Hourrègue, Julien Dumurgier, Lisette Volpe-Gillot, Claire Paquet

**Affiliations:** 1 Université de Paris, INSERM U1144 Optimisation Thérapeutique en Neuropsychopharmacologie, Paris, France; 2 Centre de Neurologie Cognitive, GHU APHP Nord Hôpital Lariboisière Fernand-Widal, Paris, France; 3 COVID Unit Féréol, AP-HP, GHU APHP Nord Hôpital Lariboisière Fernand-Widal, 200 rue du Faubourg Saint-Denis, 75010 Paris, France; 4 Unité Neuropsychogériatrique, Léopold Bellan Hôpital, Paris, France; 5 Hôpital Charles Foix, Department of Geriatric Medicine, APHP, Ivry sur Seine, France; 6 Département de Psychiatrie et de Médecine Addictologique, GHU APHP Nord Hôpital Lariboisière Fernand-Widal, Paris, France; 7 Service de Gériatrie, GHU APHP Nord Hôpital Lariboisière Fernand-Widal, Paris, France

**Keywords:** COVID-19, SARS-CoV-2, Dementia, Mortality, Prognostic factors

## Abstract

**Background:**

There is limited evidence on the characteristics and outcome of patients with dementia hospitalised for novel coronavirus infection (COVID-19).

**Method:**

We conducted a prospective study in 2 gerontologic COVID units in Paris, France, from March 14, 2020, to May 7, 2020. Patients with dementia hospitalised for confirmed COVID-19 infection were systematically enrolled. A binary logistic regression analysis was performed to identify factors associated with mortality at 21 days.

**Results:**

We included 125 patients. Median age was 86 (IQI 82–90); 59.4% were female. Most common causes of dementia were Alzheimer’s disease, mixed dementia and vascular dementia. 67.2% had ≥ 2 comorbidities; 40.2% lived in a long-term care facility. The most common symptoms at COVID-19 onset were confusion and delirium (82.4%), asthenia (76.8%) and fever (72.8%) before polypnea (51.2%) and desaturation (50.4%). Falls were frequent at the initial phase of the disease (35.2%). The fatality rate at 21 days was 22.4%. Chronic kidney disease and CRP at admission were independent factors of death. Persisting confusion, mood and behavioural disorders were observed in survivors (19.2%).

**Conclusion:**

COVID-19 in demented individuals is associated with severe outcome in SARS-CoV-2 infection and is characterised by specific clinical features and complications, with confusion and delirium at the forefront. COVID-19 testing should be considered in front of any significant change from baseline.

## Introduction

On January 30, 2020, the World Health Organization (WHO) drew attention to a new coronavirus disease 2019 (COVID-19), declaring it a public health emergency of international concern. As of February 4, 2021, there had been more than 100 million of cases and 2 million deaths worldwide. The clinical spectrum of COVID-19 infection appears broad, encompassing asymptomatic infections, mild upper respiratory tract illness and severe pneumonia with respiratory failure, systemic complications and multi-organ failure [1].

Severe COVID-19 affects elderly with chronic diseases, including cognitive decline, in high proportion compared to the general adult population [2, 3]. According to recent studies, dementia is a major risk factor for COVID-19 severity [4, 5]. Concomitantly, the risk of exposure to the infection is more important in patients with dementia, highly exposed in the context of long-term care facilities, frequent hospitalizations and intellectual decline. So far, specific clinical features and prognostic factors of COVID-19 in demented patients remain unclear [6, 7].

In order to identify specific features and risk factor of death in demented people, we report a cohort study on 125 patients with dementia hospitalised for a confirmed COVID-19 infection.

## Methods

### Study design

This prospective cohort study systematically included patients over 65 with dementia hospitalised for COVID-19 infection in two centres: GHU APHP Nord Hôpital Lariboisière Fernand Widal Université de Paris and Leopold Bellan Hospital, Paris, between March 14 and May 7, 2020. The definite diagnosis of COVID-19 was determined through reverse-transcription polymerase chain reaction (RT-PCR) testing of pharyngeal swabs or chest CT. We collected demographics, medical history, clinical presentation, laboratory results, treatment, complications and outcome. The outcome was death at 21 days, to assess short-term case fatality rate. Follow-up period was determined upon the reported length of disease to death in the literature [8], median delay of evolution to ARDS, described delay of viral shedding in survivors [3] and mean duration of patient’s hospitalisation [2]. Dementia was defined according to the American Psychiatric Association’s Diagnostic and Statistical Manual (DSM-5) as a significant cognitive decline from a previous level of performance in one or more cognitive domains interfering with independence in everyday activities [9]. Aetiology of the dementia was collected when available. Comorbidities were evaluated using the Charlson Index [10]. Blood oxygen desaturation was defined by a saturation under 93% or a loss of 3% or more. Acute kidney injury was diagnosed according to KDIGO definition [11]. Cardiac injury was diagnosed clinically or throughout abnormalities observed on electrocardiography and serum level of troponin and Brain natriuretic Peptide (BNP). COVID-19 severity and acute respiratory distress syndrome were defined following the WHO recommendations. Transfer in intensive care unit (ICU) in case of clinical deterioration was discussed at admission with the physician in charge, ICU doctors, palliative care team and patients and caregivers, and the decision of a potential transfer was documented in the patient written file.

### Statistical analysis

Descriptive data are shown as median (interquartile interval) or percentage (number of subjects). Chi-square test or Fisher’s test was used to compare qualitative data between groups. Mann-Whitney test and Kruskal-Wallis were applied to analyse non-normally distributed data. A binary logistic regression analysis was performed to identify clinical and demographic characteristics associated with mortality. We included the variables that had differed in descriptive analysis between survivors and non-survivors. Variables with missing data were excluded from this analysis. A two-tailed *p* value < 0.05 was considered significant. Statistical analysis was performed using GraphPad Prism and SPSS 85 Statistics version 26.0.

*Ethical statement:* we obtained the required approval from the Commission Nationale Informatique et Liberté (CNIL) to collect anonymised data.

## Results

We included a total of 125 patients. Patients’ demographic and clinical characteristics are summarised in Table 1. The median age was 86 (82–91) years, and 41.6% of included subjects were male. All patients had a clinical diagnosis of dementia. 40% of the patients had received a specific diagnosis: most frequent causes of dementia were Alzheimer’s disease (AD) (10.4%), vascular dementia (7.2%), and multiple aetiologies dementia (7.2%). Parkinson’s disease and atypical parkinsonian syndromes, fronto-temporal dementia, alcohol-related cognitive impairment and psychosis accounted for other diagnoses. The other patients (60%) presented with unexplored major cognitive impairment. Patients frequently presented with associated mood disorder (31.2%) and psychotic symptoms (12%). A stroke history was noted in 32% of patients. A former history of fall was reported for 38.4% of patients. Sixty per cent of patients received psychotropic treatment: antidepressants/mood regulators (35.2%), anxiolytic (30.4%) and neuroleptics (12.0%). Most patients were living at home (59.2%) while 40.8% were living in a long-term care facility.

**Table 1:** Characteristics of included patients

	Total (*n* = 125)	Survivors (*n* = 97)	Non-survivors (*n* = 28)	*p*
Age	86 (82–91)	87 (82–91)	86 (82–93)	0.815
Male sex	41.6% (52)	36.1% (35)	60.7% (17)	**0.029**
Admission from:				0.663
Home	59.2% (74)	57.7% (56)	64.3% (18)	
Long-term care facility	40.8% (51)	42.3% (41)	35.7% (10)	
Medical history				
Comorbidities	91.2% (114)	91.8% (89)	89.3% (25)	0.709
> 2 Comorbidities	67.2% (84)	67.0% (65)	67.9% (19)	0.822
Charlson Comorbidity Index	6 (5–7)	6 (5–7)	6 (5–7)	0.292
Neurological status				
*Etiologies of dementia*				
Unspecified cognitive impairment	60.0% (75)	59.8% (58)	60.7% (17)	1.000
Diagnosed dementia	40.0% (50)	40.2% (39)	39.3% (11)	1.000
Alzheimer’s disease	10.4% (13)	11.3% (11)	7.1% (2)	0.731
Vascular dementia	7.2% (9)	6.2% (6)	10.7% (3)	0.418
Other causes of dementia	7.2% (9)	5.2% (5)	14.3% (4)	0.113
Parkinson disease, parkinsonian syndromes	9.6% (12)	10.3% (10)	7.1% (2)	1.000
Psychosis	3.2% (4)	4.1% (4)	0.0% (0)	1.000
Alcohol-related dementia	2.4% (3)	3.1% (3)	0.0% (0)	1.000
*Neurological symptoms and history*				
Mood disorder	31.2% (39)	33.0% (32)	25.0% (7)	0.640
Psychotic symptoms	12.0% (15)	12.4% (12)	10.7% (3)	1.000
History of stroke	32.0% (40)	32.0% (31)	32.1% (9)	1.000
History of fall	38.4% (48)	36.1% (35)	46.4% (13)	0.373
Walking before admission	70.4% (88)	70.1% (68)	71.4% (20)	1.00
Psychotropic treatment	60.0% (75)	60.8% (59)	57.1% (16)	0.826
Anxiolytic	30.4% (38)	32.0% (31)	25.0% (7)	0.642
Antidepressant, mood regulator	35.2% (44)	35.1% (34)	35.7% (10)	1.000
Neuroleptics	12.0% (15)	11.3% (11)	14.3% (4)	0.742
Non-neurological comorbidities				
Cardiovascular disease	40.8% (51)	38.1% (37)	50.0% (14)	0.383
Arterial hypertension	80.0% (100)	79.4% (77)	82.1% (23)	1.00
Hypercholesterolaemia	48.8% (61)	46.4% (45)	57.1% (16)	0.392
Atrial fibrillation	24.8% (31)	24.7% (24)	25.0% (7)	1.00
Coronaropathy	16.0% (20)	14.4% (14)	21.4% (6)	0.388
Cardiac insufficiency	24.0% (30)	25.8% (25)	17.9% (5)	0.460
Thrombosis	9.6% (12)	11.3% (11)	3.6% (1)	0.296
COPD	8.8% (11)	6.2% (6)	17.9% (5)	0.068
Respiratory insufficiency	3.2% (4)	3.1% (3)	3.6% (1)	1.000
Diabetes	16.8% (21)	15.5% (15)	21.4% (6)	0.566
Malnutrition	33.6% (42)	37.1% (36)	21.4% (6)	0.172
Malignancy	9.6% (12)	11.3% (11)	3.6% (1)	0.296
Chronic liver disease	1.6% (2)	2.1% (2)	0.0% (0)	1.000
Chronic renal disease	16.0% (20)	11.3% (11)	32.1% (9)	**0.017**

Data are median (IQI), % (*n*). *P* values > 0.05 (bold) indicates significant differences between survivors and non-survivors

*Other causes of dementia: included mixed dementia (AD and vascular dementia, *n* = 7), fronto-temporal dementia (*n* = 1) and vascular dementia and alcohol-related dementia (respectively, *n* = 1)

*COPD* chronic obstructive pulmonary disease

Non-neurological comorbidities were frequent (91.2%) with 67.2% of patients with 3 or more comorbidities. Median Charlson comorbidities index was 6 (5–7). Most frequent comorbidities were hypertension (80%), dyslipidaemia (48.8%) and history of cardiovascular disease (40.8%).

### Clinical presentation and biological findings

Infection was confirmed in 93.2% of patients by PCR test and in 6.8% by chest CT (Table 2). The most common initial symptoms were confusion and delirium observed in 82.4% of patients. It was notably the only symptom at onset and during the evolution of the disease for three patients in our cohort, thus COVID-19 infection was screened for considering the pandemic context. The most frequent associated general symptoms were asthenia (76.8%), fever (72.8%) and anorexia (56.8%). Most frequent respiratory symptoms were polypnea (51.2%), desaturation (50.4%) and cough (49.6%). An initial fall was reported in 35.2%. No patient complained of loss of taste or smell.

**Table 2:** COVID clinical symptoms and laboratory results

Symptoms at admission	Total (*n* = 125)	Survivors (*n* = 97)	Non-survivors (*n* = 28)	*P*
Fever	72.8% (91)	71.1% (69)	78.6% (22)	0.482
Cough	49.6% (62)	51.5% (50)	42.9% (12)	0.521
Expectorations	21.6% (27)	18.6% (18)	32.1% (9)	0.190
Polypnea	51.2% (64)	42.3% (41)	82.1% (23)	**< 0.0001**
Desaturation	50.4% (63)	43.3% (42)	75.0% (21)	**0.002**
Minimum saturation	90 (88–92)	90 (88–92)	90 (88–91)	0.167
Digestive symptoms	16.8% (21)	18.6% (18)	10.7% (3)	0.400
Asthenia	76.8% (96)	72.2% (70)	92.9% (26)	**0.023**
Myalgia	15.2% (19)	15.5% (15)	14.3% (4)	1.000
Anorexia	56.8% (71)	53.6% (52)	67.9% (19)	0.201
Headache	4.0% (5)	4.1% (4)	3.6% (1)	0.688
Confusion, delirium	82.4% (103)	81.4% (79)	85.7% (24)	0.780
Initial fall	35.2% (44)	33.0% (32)	42.9% (12)	0.373
Laboratory findings				
Haemoglobin, g/dL	12.1 (10.9–13.08)	12.2 (10.9–13.3)	11.6 (10.3–12.7)	0.152
Lymphocytes count (mm^3^)	0.99 (0.73–1.29)	1.0 (0.8–1.3)	0.9 (0.6–1.2)	**0.032**
Lymphopaenia, %	84.8% (106)	85.6% (83)	82.1% (23)	0.544
White blood cell count (mm^3^)	5.8 (3.9–7.9)	5.4 (3.9–7.1)	6.2 (4.1–8.7)	0.070
Platelets count (mm^3^)	207 (164.3–265)	210 (170–261)	202 (150–283)	0.995
Thrombopaenia, %	16.0% (20)	13.4% (13)	25.0% (7)	0.146
Initial C-reactive protein, mg/dL	30.85 (8.75–84.75)	25.8 (8.0–64.5)	76.0 (33.0–44.0)	**< 0.0001**
Initial creatinine, μmol/L	78 (64–103.8)	74.0 (63.5–94.0)	127.0 (70.0–159.0)	**0.003**
Aspartate aminotransferase, U/L	36 (24–54)	35 (24–53)	46 (32–61)	0.053
Alanine aminotransferase, U/L	18 (13–31)	17.00 (13.00–31.00)	23.50 (12.75–34.75)	0.438
Albumin, g/L	30 (26–34)	30.8 (27–34)	28.5 (23–33)	0.088
Troponin positivity, %^#^	35.5% (22)	30.4% (14)	50.0% (8)	**0.032**

Data are presented as median (IQI), % (*n*). *P* values > 0.05 (bold) indicates significant differences between survivors and non-survivors. ^#^Troponin was available for 62 subjects

The most common biochemical abnormality was lymphopaenia (84.8%) with a median value of lymphocytes of 0.99 (0.73–1.29), increased CRP at admission and low albumin. Thrombopaenia was observed in 16% of cases. Troponin was available for 22 patients and increased in 35.5% of patients.

### Treatment and outcome

Treatment and outcome are reported in Table 3. Persisting behavioural disorders during hospitalisation were observed in 19.2% of subjects. Other neurological complications included strokes (2.4%, 3 patients) and seizures (2.4%, 3 patients). Treatment mostly associated oxygen therapy (60%) and antibiotics (61.6%). A minority of patients received specific treatment for COVID-19 (hydroxychloroquine 16.8% and corticosteroids 8.0%). Acute respiratory distress syndrome (ARDS) occurred in 25.6% of patients. Associated complications were bacterial superinfection (19.2%), acute kidney injury (16.8%), cardiac failure (13.6%) and diabetic acidoketosis (2.4%). Treatment by neuroleptics or antidepressant was not associated to outcome.

**Table 3:** Treatment, complications and outcome

	Total (*n* = 125)	Survivors (*n* = 97)	Non-survivors (*n* = 28)	*P*
Covid severity*				**0.001**
Mild	37.6% (47)	48.5% (47)	0.0% (0)	
Moderate	27.2% (34)	35.0% (34)	0.0% (0)	
Severe	35.2% (44)	16.5% (16)	100% (28)	
Treatment				
Oxygen therapy	60.0% (75)	50.5% (49)	92.9% (26)	**< 0.0001**
Maximal flow (L/min)	3.0 (2.0–6.0)	3.0 (2.0–6.0)	5.0 (2.5–15.0)	**0.018**
Antibiotics use	61.6% (77)	57.7% (56)	75.0% (21)	0.074
Combination of antibiotics	33.6% (42)	29.9% (29)	46.4% (13)	0.082
Beta-lactamine	54.4% (68)	50.5% (49)	67.9% (19)	0.079
Macrolides	37.6% (47)	35.1% (34)	46.4% (13)	0.279
2nd line antibiotics	17.6% (22)	14.4% (14)	28.6% (8)	0.096
Steroids	8.0% (10)	8.2% (8)	7.1% (2)	1.000
Hydroxychloroquine	16.8% (21)	16.5% (16)	17.9% (5)	1.000
Neuroleptics	4.8% (6)	5.2% (5)	3.6% (1)	0.678
Complications				
ARDS	25.6% (32)	6.2% (6)	92.9% (26)	**< 0.0001**
Cardiac injury	13.6% (17)	9.3% (9)	28.6% (8)	**0.024**
ACKI	16.8% (21)	12.4% (12)	32.1% (9)	**0.021**
Bacterial superinfection	14.4% (18)	11.3% (11)	25.0% (7)	0.122
Behavioural disorder	19.2% (24)	21.6% (21)	10.7% (3)	0.278
Diabetic ketoacidosis	2.4% (3)	3.1% (3)	0.0% (0)	1.000
Seizure	2.4% (3)	2.1% (2)	3.6% (1)	0.536
Stroke	2.4% (3)	2.1% (2)	3.6% (1)	1.000
Outcome	Total (*n*=125)	Survivors (*n*=97)	Non-survivors (*n*=28)	
Total mortality	22.4% (28)			
Delay admission-death			8.5 (3.7–16)	
Length of stay		16 (11–19.7)		
Discharge home before end of follow-up		40.2% (39)		

Data are median (IQI), % (*n*). *P* values > 0.05 (bold) indicates significant differences between survivors and non-survivors

*COVID severity defined according to WHO guidelines

*ACKI* acute kidney injury, *ARDS* acute respiratory distress syndrome

Mortality at 21 days was 22.4%. The large majority of deaths (92.9%) was attributed to ARDS. Cardiac failure and osmolar coma were associated causes of death. Non-survivors were overrepresented in men (60.7% versus 36.1% in survivors, *P* = 0.029), presenting more frequent chronic kidney disease. Respiratory distress, lower lymphocytes count, high CRP and positive troponin were more frequent in non-survivors. No statistically significant difference was observed in the rate of death between patients with a diagnosed neurodegenerative disorder compared to the patients with unexplored cognitive impairment (*P* = 1.000) or in those living in long-term facilities (*P* = 0.663).

Male sex, chronic kidney disease, desaturation, dyspnoea as well as lymphocytes count, CRP and serum creatinine at admission were included in a multivariate binary logistic regression model to identify associated factors of death (Table 4). Two variables were independently associated to death at 21 days: CRP at admission (OR = 1.013, *P* = 0.004) and chronic kidney disease (OR = 4.631, *P* = 0.025).

**Table 4:** Predictive factors of mortality at 21 days of diagnosis

	Exp(B)	95% CI for EXP(B)		*P*
		Lower	Upper	
Male sex	1.120	0.379	3.310	0.838
Chronic renal disease	4.631	1.215	17.649	**0.025**
Desaturation	1.879	0.418	8.452	0.411
Dyspnoea	2.100	0.452	9.765	0.344
Lymphocytes	1.096	0.649	1.852	0.732
Creatinine	0.999	0.992	1.007	0.850
CRP	1.013	1.004	1.021	**0.004**

Multivariate binary logistic regression for identifying predictive factors of outcome. Troponin was excluded of the analysis due to missing data. *P* < 0.05 indicates significance (bold)

*95% CI* 95% confidence interval, *CRP* C-reactive protein

## Discussion

In this study, we aimed at describing specific clinical features and prognostic factors of mortality in demented patients. Confusion with or without general symptoms is the most frequent initial presentation and was not a predictive factor of death while history of chronic kidney disease and CRP level at admission were significantly associated with mortality.

Studies focusing on the neurological features of COVID-19 suggest that confusion occurred in 20–30% of hospitalised patients increasing to 60–70% in severe forms [12, 13]. Older adults are more prone to experiencing confusion and delirium and dementia is the higher predisposing risk factor (OR from 2.3–4.7), before age, visual and hearing impairment and polypharmacy [14, 15]. Several series and cases reports highlight COVID-19 infection presenting as isolated and persistent confusion [16–19] and as a factor of negative outcome [20]. In our series, confusion and delirium occurred independently of the severity of the COVID-19 infection and did not appear as a factor of negative outcome. The discrepancy between all these results could be explained by the high prevalence of confusion in our cohort that might have not allowed to study his weight on prognosis.

Overall, the prevalence of confusion and delirium was higher than the one reported in older adults admitted with non-COVID-19 pneumonia [21, 22] and in patients admitted with severe acute respiratory syndrome (SARS) and Middle East respiratory syndrome (MERS) [23].

The pathophysiological explanations remain unclear. COVID-19 could have a direct neuronal toxicity through CNS invasion [24]. Cognitive symptoms could also indirectly be related to neuroinflammation, corresponding to “sickness behaviour” to which demented subjects have been shown to present with increased vulnerability [25]. An increase of neurofilaments lights and glial fibrillary acid protein, respectively reflecting neuronal injury and glial activation, has been observed in patients with moderate and severe COVID-19 with or without dementia [26]. The neuronal and synaptic fragility in demented patients may be particularly prone to injury induced by COVID-19, either through direct infectious lesion or through indirect inflammatory mechanisms. Another frequently reported neurologic symptom in our series is falls, as already reported [5, 27]. General risk of fall is high in demented older adults; gait impairment and falls are more prevalent in dementia than in normal ageing and are related to the severity of cognitive impairment [28]. Rupture of homeostasis in the context of viral infection would account for their frequent occurrence [29].

Besides confusion, delirium and falls, a broad range of neurological complications, caused directly or indirectly by the virus, including infectious, para-infectious, and post-infectious encephalitis, stroke related to coagulopathy, and acute neuropathies have been reported [30].

Stroke and seizure were observed during disease evolution for respectively 3 of our patients, at a similar rate as the one observed in cases of a cohort of around 5000 subjects [31]. No patient reported olfactory or gustatory symptom in our cohort. Previous olfactory dysfunction could partially account for this finding as hypo- and anosmia are associated to alpha-synucleinopathies and more generally to cognitive decline in older adults [32]. Moreover, studies on COVID-19 have reported a lower prevalence of olfactory and gustatory symptoms in older patients and severe forms of the disease [33, 34].

Case fatality rate at 21 days was 22.4%, in line with previous findings in large cohorts [2, 5]. A study by Canavelli et al. [35] evaluating the prevalence of dementia in a random sample of confirmed COVID-19-infected patients, found that patients with dementia accounted for 15.8% of overall COVID-19-related death. In a meta-analysis, the mortality of individuals with dementia was increased compared to subjects with no cognitive impairment (OR = 5.17) [36]. More specifically, Matias-Giu et al. have shown that AD patients showed a higher risk of death in COVID-19 than patients with fronto-temporal dementia. One explanation could be that the APOE e4 genotype (the highest risk factor for AD) has been reported as a predictive factor of severe COVID-19 [20] and death [21]. In our series, we did not find any difference between patients with a diagnosed dementia (AD, vascular dementia, alcohol-related cognitive impairment) and patients with unexplored cognitive impairment.

History of chronic kidney disease and high CRP at admission were independently associated with death. Our overall data are in accordance with previous reports in which male sex, multiple comorbidities, elevated CRP and low lymphocyte count were observed in the majority of COVID-19 deaths [3, 37]. None of our patients who underwent ARDS was deemed suitable candidate for ICU or invasive ventilation in regard to clinical status after multidisciplinary decision. Patients received variable treatment, associating antibiotics, rarely antiviral treatment, high flow oxygen and corticosteroids or immunomodulatory drugs for a small number. The fast-changing evidence regarding treatment of COVID 19, the variability of comorbidities and frequent contraindications can explain the variability in treatment reported in the cohort. Given the size of our cohort, we were not able to assess the efficiency or draw any recommendation. Therapeutic essays have been ongoing regarding the potential beneficial effect of serotonin reuptake inhibitor (SSRI) through modulation inflammatory response during sepsis [38]; no difference in outcome was observed regarding treatment by antidepressants in our cohort.

All in all, in the actual context of a second wave of COVID-19, this work demonstrates that special attention should be given to demented patients who manifest confusion with acute behavioural changes and falls, with or without asthenia, fever and lymphopaenia.

### Limitations

This study has several limitations. Our studied population was drawn from geriatric hospitalised samples of demented patients in need of hospital care, limiting its generalisation. Patients presenting with atypical symptoms and lacking respiratory symptoms or fever may not have been identified as COVID cases and included in our analysis. Due to low group numbers, we could not assess the difference in severity and mortality between the different etiologies of dementia and no patient of our cohort was admitted in ICU. We did not dispose of a control group of older adults without cognitive impairment. Also, we focused on short-term mortality; mid- and long-term follow-up could allow us to identify additional prognostic factors.

## Conclusion

In our cohort of individuals with dementia, we have observed that COVID-19 was characterised by atypical presentation with prevalent nonspecific symptoms at the initial phase of the disease, namely persisting confusion and behavioural disorders as well as frequent falls. Early recognition of COVID-19 in demented adults should help provide early treatment and adequate care and isolation and COVID-19 testing should be considered in front of any significant change from baseline.

## Data Availability

The full datasets used during the current study are available from the corresponding author on reasonable request.

## References

[1] Wu Z, McGoogan JM (2020). Characteristics of and important lessons from the coronavirus disease 2019 (COVID-19) outbreak in China: summary of a report of 72 314 cases from the Chinese Center for Disease Control and Prevention. JAMA.

[2] Richardson S, Hirsch JS, Narasimhan M, Crawford JM, McGinn T, Davidson KW, Barnaby DP, Becker LB, Chelico JD, Cohen SL, Cookingham J, Coppa K, Diefenbach MA, Dominello AJ, Duer-Hefele J, Falzon L, Gitlin J, Hajizadeh N, Harvin TG, Hirschwerk DA, Kim EJ, Kozel ZM, Marrast LM, Mogavero JN, Osorio GA, Qiu M, Zanos TP, and the Northwell COVID-19 Research Consortium (2020). Presenting characteristics, comorbidities, and outcomes among 5700 patients hospitalized with COVID-19 in the New York City area. JAMA.

[3] Zhou F, Yu T, Du R, Fan G, Liu Y, Liu Z (2020). Clinical course and risk factors for mortality of adult inpatients with COVID-19 in Wuhan, China: a retrospective cohort study. Lancet.

[4] Martín-Jiménez P, Muñoz-García MI, Seoane D, Roca-Rodríguez L, García-Reyne A, Lalueza A, Maestro G, Folgueira D, Blanco-Palmero VA, Herrero-San Martín A, Llamas-Velasco S, Pérez-Martínez DA, González-Sánchez M, Villarejo-Galende A (2020). Cognitive impairment is a common comorbidity in deceased COVID-19 patients: a hospital-based retrospective cohort study. J Alzheimers Dis.

[5] Atkins JL, Masoli JAH, Delgado J, Pilling LC, Kuo C-L, Kuchel GA, Melzer D (2020). Preexisting comorbidities predicting COVID-19 and mortality in the UK biobank community cohort. J Gerontol A Biol Sci Med Sci.

[6] Matias-Guiu JA, Pytel V, Matías-Guiu J (2020). Death rate due to COVID-19 in Alzheimer’s disease and frontotemporal dementia. J Alzheimers Dis.

[7] Bianchetti A, Rozzini R, Guerini F, Boffelli S, Ranieri P, Minelli G, Bianchetti L, Trabucchi M (2020). Clinical presentation of COVID19 in dementia patients. J Nutr Health Aging.

[8] Karagiannidis C, Mostert C, Hentschker C, Voshaar T, Malzahn J, Schillinger G, Klauber J, Janssens U, Marx G, Weber-Carstens S, Kluge S, Pfeifer M, Grabenhenrich L, Welte T, Busse R (2020). Case characteristics, resource use, and outcomes of 10 021 patients with COVID-19 admitted to 920 German hospitals: an observational study. Lancet Respir Med.

[9] American Psychiatric Association, American Psychiatric Association (2013). Diagnostic and statistical manual of mental disorders: DSM-5.

[10] Charlson ME, Pompei P, Ales KL, MacKenzie CR (1987). A new method of classifying prognostic comorbidity in longitudinal studies: development and validation. J Chronic Dis.

[11] Khwaja A (2012). KDIGO clinical practice guidelines for acute kidney injury. Nephron Clin Pract.

[12] Helms J, Kremer S, Merdji H, Clere-Jehl R, Schenck M, Kummerlen C, Collange O, Boulay C, Fafi-Kremer S, Ohana M, Anheim M, Meziani F (2020). Neurologic features in severe SARS-CoV-2 infection. N Engl J Med.

[13] Garcez FB, Aliberti MJR, Poco PCE, Hiratsuka M, Takahashi S de F, Coelho VA, et al. Delirium and adverse outcomes in hospitalized patients with COVID-19. J Am Geriatr Soc 2020. doi: 10.1111/jgs.16803. 10.1111/jgs.16803 PMC7460960 32835425

[14] Witlox J, Eurelings LSM, de Jonghe JFM, Kalisvaart KJ, Eikelenboom P, van Gool WA (2010). Delirium in elderly patients and the risk of postdischarge mortality, institutionalization, and dementia: a meta-analysis. JAMA.

[15] Inouye SK, Westendorp RGJ, Saczynski JS (2014). Delirium in elderly people. Lancet.

[16] Alkeridy WA, Almaglouth I, Alrashed R, Alayed K, Binkhamis K, Alsharidi A (2020). A unique presentation of delirium in a patient with otherwise asymptomatic COVID-19. J Am Geriatr Soc.

[17] Tay HS, Harwood R (2020). Atypical presentation of COVID-19 in a frail older person. Age Ageing.

[18] Butt I, Sawlani V, Geberhiwot T (2020). Prolonged confusional state as first manifestation of COVID-19. Ann Clin Transl Neurol.

[19] Vrillon A, Hourregue C, Azuar J, Grosset L, Boutelier A, Tan S, Roger M, Mourman V, Mouly S, Sène D, François V, Dumurgier J, Paquet C. J Am Geriatr Soc. 2020;68(12):2735–43. 10.1111/jgs.16894 PMC7675559 33045106

[20] Poloni TE, Carlos AF, Cairati M, Cutaia C, Medici V, Marelli E, Ferrari D, Galli A, Bognetti P, Davin A, Cirrincione A, Ceretti A, Cereda C, Ceroni M, Tronconi L, Vitali S, Guaita A (2020). Prevalence and prognostic value of delirium as the initial presentation of COVID-19 in the elderly with dementia: an Italian retrospective study. EClinicalMed.

[21] Pieralli F, Vannucchi V, Mancini A, Grazzini M, Paolacci G, Morettini A, Nozzoli C (2014). Delirium is a predictor of in-hospital mortality in elderly patients with community acquired pneumonia. Intern Emerg Med.

[22] Aliberti S, Bellelli G, Belotti M, Morandi A, Messinesi G, Annoni G, Pesci A (2015). Delirium symptoms during hospitalization predict long-term mortality in patients with severe pneumonia. Aging Clin Exp Res.

[23] Rogers JP, Chesney E, Oliver D, Pollak TA, McGuire P, Fusar-Poli P, Zandi MS, Lewis G, David AS (2020). Psychiatric and neuropsychiatric presentations associated with severe coronavirus infections: a systematic review and meta-analysis with comparison to the COVID-19 pandemic. Lancet Psychiatry.

[24] Aghagoli G, Gallo Marin B, Katchur NJ, Chaves-Sell F, Asaad WF, Murphy SA. Neurological involvement in COVID-19 and potential mechanisms: a review. Neurocrit Care. 2020:1–10. 10.1007/s12028-020-01049-4. 10.1007/s12028-020-01049-4 PMC7358290 32661794

[25] Holmes C, Cunningham C, Zotova E, Culliford D, Perry VH (2011). Proinflammatory cytokines, sickness behavior, and Alzheimer disease. Neurology.

[26] Kanberg N, Ashton NJ, Andersson L-M, Yilmaz A, Lindh M, Nilsson S, Price RW, Blennow K, Zetterberg H, Gisslén M (2020). Neurochemical evidence of astrocytic and neuronal injury commonly found in COVID-19. Neurology.

[27] Norman RE, Stall NM, Sinha SK (2020). Typically atypical: COVID-19 presenting as a fall in an older adult. J Am Geriatr Soc.

[28] van Iersel MB, Hoefsloot W, Munneke M, Bloem BR, Olde Rikkert MGM (2004). Systematic review of quantitative clinical gait analysis in patients with dementia. Z Gerontol Geriatr.

[29] Jensen J, Lundin-Olsson L, Nyberg L, Gustafson Y (2002). Falls among frail older people in residential care. Scand J Public Health.

[30] Ellul MA, Benjamin L, Singh B, Lant S, Michael BD, Easton A, Kneen R, Defres S, Sejvar J, Solomon T (2020). Neurological associations of COVID-19. Lancet Neurol.

[31] Frontera JA, Sabadia S, Lalchan R, Fang T, Flusty B, Millar-Vernetti P, Snyder T, Berger S, Yang D, Granger A, Morgan N, Patel P, Gutman J, Melmed K, Agarwal S, Bokhari M, Andino A, Valdes E, Omari M, Kvernland A, Lillemoe K, Chou SHY, McNett M, Helbok R, Mainali S, Fink EL, Robertson C, Schober M, Suarez JI, Ziai W, Menon D, Friedman D, Friedman D, Holmes M, Huang J, Thawani S, Howard J, Abou-Fayssal N, Krieger P, Lewis A, Lord AS, Zhou T, Kahn DE, Czeisler BM, Torres J, Yaghi S, Ishida K, Scher E, de Havenon A, Placantonakis D, Liu M, Wisniewski T, Troxel AB, Balcer L, Galetta S (2020). A prospective study of neurologic disorders in hospitalized COVID-19 patients in New York City. Neurology.

[32] Dintica CS, Marseglia A, Rizzuto D, Wang R, Seubert J, Arfanakis K, Bennett DA, Xu W (2019). Impaired olfaction is associated with cognitive decline and neurodegeneration in the brain. Neurology.

[33] Porta-Etessam J, Núñez-Gil IJ, González García N, Fernandez-Perez C, Viana-Llamas MC, Eid CM, Romero R, Molina M, Uribarri A, Becerra-Muñoz VM, Aguado MG, Huang J, Rondano E, Cerrato E, Alfonso E, Mejía AFC, Marin F, Roubin SR, Pepe M, Feltes G, Maté P, Cortese B, Buzón L, Mendez JJ, Estrada V. COVID-19 anosmia and gustatory symptoms as a prognosis factor: a subanalysis of the HOPE COVID-19 (Health Outcome Predictive Evaluation for COVID-19) registry. Infection. 2021:1–8. 10.1007/s15010-021-01587-9. 10.1007/s15010-021-01587-9 PMC7917537 33646505

[34] Lechien JR, Chiesa-Estomba CM, Vaira LA, De Riu G, Cammaroto G, Chekkoury-Idrissi Y, et al. Epidemiological, otolaryngological, olfactory and gustatory outcomes according to the severity of COVID-19: a study of 2579 patients. Eur Arch Otorhinolaryngol. 2021. 10.1007/s00405-020-06548-w. 10.1007/s00405-020-06548-w PMC7811338 33452919

[35] Canevelli M, Palmieri L, Raparelli V, Lo Noce C, Colaizzo E, Tiple D, Vaianella L, Vanacore N, Brusaferro S, onder G, the Italian National Institute of Health COVID‐19 Mortality Group (2020). Prevalence and clinical correlates of dementia among COVID-19-related deaths in Italy. Alzheimers Dement (Amst).

[36] Liu N, Sun J, Wang X, Zhao M, Huang Q, Li H (2020). The impact of dementia on the clinical outcome of COVID-19: a systematic review and meta-analysis. J Alzheimers Dis.

[37] Cummings MJ, Baldwin MR, Abrams D, Jacobson SD, Meyer BJ, Balough EM, Aaron JG, Claassen J, Rabbani LRE, Hastie J, Hochman BR, Salazar-Schicchi J, Yip NH, Brodie D, O'Donnell MR (2020). Epidemiology, clinical course, and outcomes of critically ill adults with COVID-19 in New York City: a prospective cohort study. Lancet.

[38] Lenze EJ, Mattar C, Zorumski CF, Stevens A, Schweiger J, Nicol GE, Miller JP, Yang L, Yingling M, Avidan MS, Reiersen AM (2020). Fluvoxamine vs placebo and clinical deterioration in outpatients with symptomatic COVID-19: a randomized clinical trial. JAMA.

